# Association between air pollution exposure and coronary heart disease hospitalization in a humid sub-tropical region of China: A time-series study

**DOI:** 10.3389/fpubh.2022.1090443

**Published:** 2023-01-11

**Authors:** Ya-Ting Feng, Cui-Feng Lang, Cong Chen, Musonye Harry Asena, Yang Fang, Ruo-Di Zhang, Ling-Qiong Jiang, Xi Fang, Yue Chen, Yi-Sheng He, Peng Wang, Hai-Feng Pan

**Affiliations:** ^1^Department of Epidemiology and Biostatistics, School of Public Health, Anhui Medical University, Hefei, China; ^2^Department of General Medicine, The First Affiliated Hospital of Anhui Medical University, Hefei, China; ^3^Teaching Center for Preventive Medicine, School of Public Health, Anhui Medical University, Anhui, China

**Keywords:** air pollutants, CHD, NO_2_, CO, O_3_, disease risk

## Abstract

**Objective:**

Emerging evidence has highlighted the possible links of environmental pollution with several cardiovascular diseases (CVDs). The current study aimed to explore the impact of short-term air pollution exposure on CHD hospitalization in Hefei.

**Methods:**

Data about the daily number of CHD admissions (from 2014 to 2021) were retrieved from the First Affiliated Hospital of Anhui Medical University. Air pollutants and meteorological data were obtained from the China Environmental Monitoring Station and the China Meteorological Data Service Center, respectively. The correlation between air pollution and CHD hospitalization was assessed using distributed lag non-linear model (DLNM) and Poisson generalized linear regression.

**Results:**

In the single-pollutant model, NO_2_, O_3_, and CO strongly correlated with CHD hospitalization rate. Specifically, exposure to NO_2_ (lag0, relative risk [RR]: 1.013, 95%CI: 1.002–1.024, per 10 μg/m^3^ increase) and CO (lag13, RR: 1.035, 95%CI: 1.001–1.071, per 1 μg/m^3^ increase) revealed a positive correlation with an increased rate of CHD hospitalization. Interestingly, O_3_ had a protective association with hospitalization of CHD (lag0, RR: 0.993, 95%CI: 0.988–0.999, per 10 μg/m^3^ increase). Similar results, to those of the single-pollutant model, were revealed following verification using two-pollutant models. Subgroup analyses indicated that young people, women, and people in hot seasons were more susceptible to NO_2_ exposure, while the elderly, women, and people in cold seasons were more susceptible to O_3_. Furthermore, the elderly were more susceptible to CO exposure.

**Conclusion:**

Overall, exposure to NO_2_ and CO increases the rate of CHD hospitalization, but exposure to O_3_ shows a protective association with the rate of CHD hospitalization. Therefore, early preventive measures against air pollutants should be applied to protect vulnerable patients with CHD.

## 1. Introduction

Coronary heart disease (CHD) is a chronic heart disease that can cause myocardial ischemia, hypoxia, or necrosis and is usually caused by coronary atherosclerosis. As a typical cardiovascular disease (CVD), CHD is the leading cause of death worldwide (1). The American Heart Association (AHA) suggests that the global number of patients with CHD is likely to double by the year 2030 (2). In terms of prevalence, CHD ranks second among the world's leading causes of death. In China, the disease has been linked to the rising trend of death in the past two decades, from 1980 to 2016, when the average annual growth rate was 9.85%. Based on all these, it is clear that CHD is an important public health issue that negatively impacts people's health and quality of life.

Over the past two decades, a lot of research has profiled arterial hypertension, dyslipidemia, and smoking as risk factors associated with the increased incidence of CHD and related mortality (3). Lifestyle and diet patterns, as common influencing factors of chronic diseases, are related to the risk of CHD. In addition, temperature, relative humidity, barometric pressure, other meteorological factors, and air pollutants are also related to the risk of CHD (4–6). It is important to note that air pollution is harmful to human health in many ways. A growing number of studies have demonstrated that short-term exposure to air pollutants can cause acute and habitual damage to the CV and respiratory and immune systems (7–9). Complex components among air pollutants can induce systemic inflammation and disturb the balance of oxidative stress and the autonomic nervous system. This may cause injury to the CV system and promote the development of atherosclerosis (10, 11), eventually causing the onset and progression of CHD or stroke.

To date, a growing number of studies have shown that short-term exposure to air pollution is related to the rate of CHD (12–15). Nevertheless, most of these studies were conducted in developed countries, where climate conditions and the level of air pollution differ, in many aspects, from those in developing countries. First, comparative studies on climate characteristics and terrain conditions show that prevention and control of air pollution are more difficult in developing countries than in developed countries such as the United States and other European countries. Influenced by the topography of the Qinghai-Tibet Plateau, the Eastern part of China has a significant downdraft in winter, and the atmospheric stratification becomes more stable with less precipitation, which is not conducive to the diffusion of pollutants and wet removal. Second, global warming causes an interdecadal weakening of the East Asian winter monsoon. The average surface wind speed decreases, which is more conducive to the accumulation of pollutants. More importantly, meteorological conditions affect the transmission and diffusion of pollutants, chemical transformation, dry and wet deposition, and other processes. Even within the same country, differences in climate type and geographical location may also lead to differences in the role of atmospheric pollutants. Regardless of these, previous studies carried out in China have mainly reported on the association between PM and hospitalization of CHD (16–18), ignoring research on other pollutants. Therefore, in the present study, we carried out a time-series analysis to explore the relationship between air pollutants and CHD hospitalization in Hefei, and to identify the possible vulnerable sub-populations and meteorological factors. In addition, we employed the DLNM model, which is a modeling framework to flexibly estimate an exposure–time–response function. This modeling framework considers both the lagged effect of exposure factors and the exposure–response non-linear relationship.

## 2. Materials and methods

### 2.1. Study area

**Figure 1:**
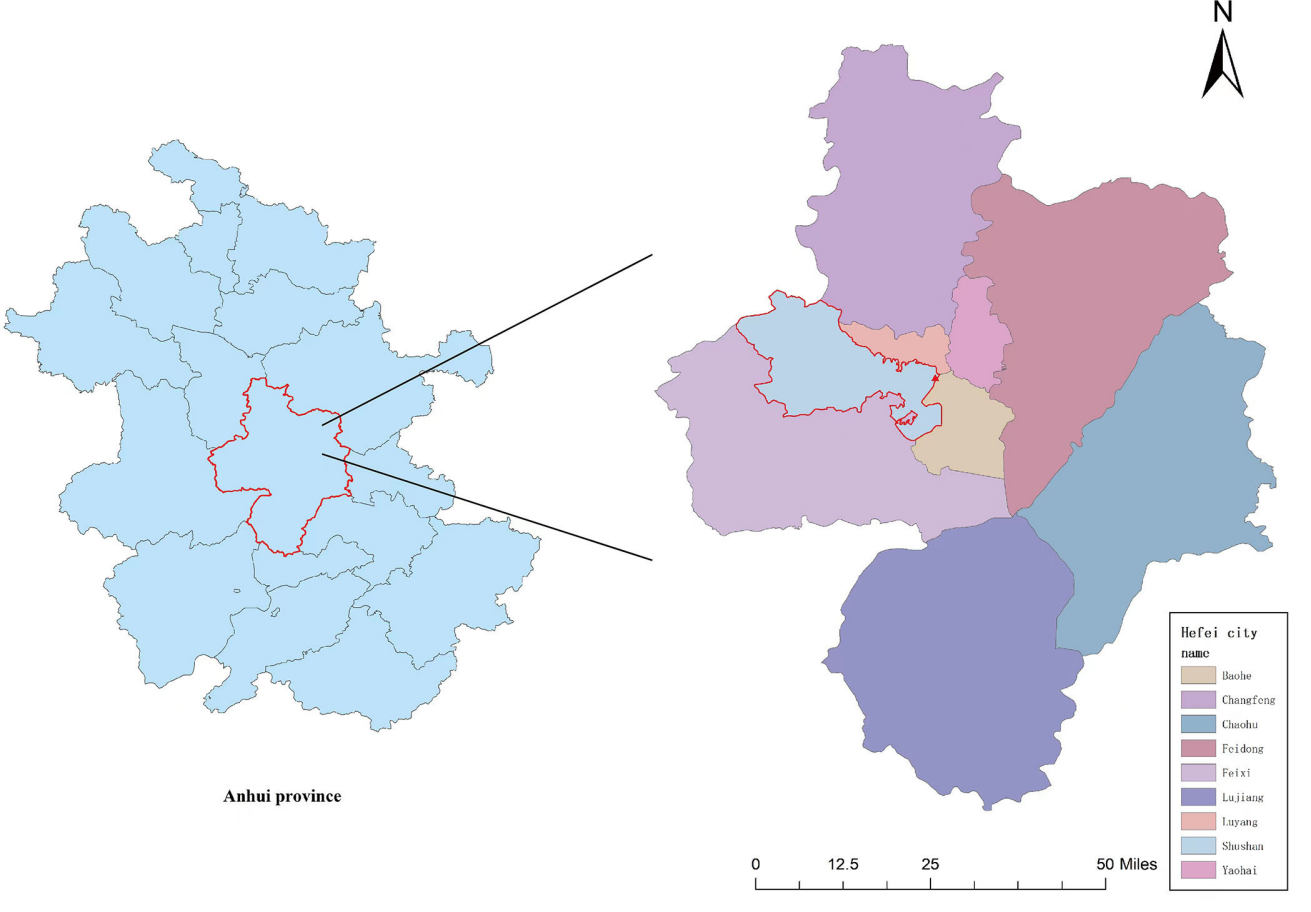
A map of the sample city and hospital (the city circled in red is Hefei, the city we studied, and the triangle represents the location of the hospital).

This study was carried out in Hefei, Anhui Province (31.87°N, 117°17E), which is among the central cities of the Yangtze River Delta region. Hefei is located in the middle latitude zone, and it is characterized by the sub-tropical monsoon humid climate conditions: obvious monsoon, distinct four seasons, relatively mild climate, and moderate annual rainfall (Figure 1).

### 2.2. Data sources

The inpatient records of CHD hospitalization were obtained from the First Affiliated Hospital of Anhui Medical University, between 1 January 2014 to 31 December 2021. The First Affiliated Hospital of Anhui Medical University is a grade-A tertiary hospital, with a bed capacity of about 5,000 beds. As the largest hospital in Hefei, the hospital's inpatient data were representative. A total of 16,656 patients were enrolled after which 10,407 patients were eliminated following the data-cleaning process. The data contained variables such as age, sex, date of hospitalization, and residential address. At the same time, to ensure that the included patients were all local, with complete basic information, patients whose permanent addresses were not in Hefei or lacked demographic information (such as age or gender) were cast aside. All admissions were diagnosed according to the definition of CHD in the 10th edition of the International Classification of Diseases (ICD-10: I25.1) (19). The Ethics Committee of Anhui Medical University approved this research.

### 2.3. Air pollutants and meteorological data

Air pollution data (NO_2_, CO, SO_2_, PM_2.5_, PM_10_, and O_3_ 8 h) from 2014 to 2021 were obtained from China's Environmental Monitoring stations. For each pollutant, we obtained the average concentration of 10 state-controlled air quality monitoring stations in Hefei. The daily data on relative humidity (RH) (%) and average temperature (MT) (°C), during the research period, were collected by the China Meteorological Data Service Center (http://data.cma.cn/).

### 2.4. Statistical analysis

The spearman analysis and the depicted scatter plot were used to determine the correlation coefficients between air pollutants, meteorological factors, and the number of CHD admissions. A distributed lag non-linear model (DLNM) was used to test the relationship between air pollution exposure and CHD hospitalization rates (20). Based on the exposure–response relationship, DLNM increases the lag effect because it considers both the lagged effect of exposure factors and the non-linear relationship of the exposure–response (21). Since CHD hospitalization is a probability event, we used the quasi-Poisson distribution generalized linear model (GLM) to explore the association between air pollutants and daily CHD hospitalization (22). In addition, this study brought the pollutants into the single pollutant model to explore the exposure–response relationship and the lag–response relationship. Since there may be possible interactions between various pollutants, the statistical correlations between pollutants were also conducted. To avoid multi-collinearity, a Spearman correlation coefficient of < 0.7 was selected as a covariate. Furthermore, to effectively control the effects of meteorological factors, long-running trends, the weekday effect, the holiday effect, and other potential confounding factors, these variables were incorporated into the control model. The quasi-Poisson Akaike Information Criterion (Q-AIC) was applied to evaluate the goodness of fit of the model and determine the lag time and degree of freedom (df). Finally, the time series model was constructed as below:

*Yt* quasi-Poisson (μ_*t*_)


(1) $$\begin{array}{l} \begin{array}{lll} \text{Log}(\mu_{\text{t}})\; & = & \;\alpha +\beta {\text{X}}_{\text{t}},\text{l}+\text{ns}(\text{RH},\text{3})+\text{ns}(\text{MT},\text{3}) \end{array}\\ \;\begin{array}{ll} + & \text{ns}(\text{Timet},{\text{7}}^{*}\text{8})+\eta {\text{DOW}}_{\text{t}}+\gamma {\text{Holiday}}_{\text{t}} \end{array} \end{array}$$

where *t* is the observation time (day), μ_*t*_ is the number of patients with CHD hospitalized on day *t*, α is the model's intercept, *X*_*t*_, *l* is the cross-basis matrix of the DLNM model, *l* is the lag day, β is the vector of *X*_*t*_, and ns (RH,3) is the natural cubic spline. In this study, a natural cubic spline of 7 dfs/year was applied to regulate seasonal and long-running trends (23). DOW is the dummy variable of a day of the week. We applied the binary variable Holiday_*t*_ to regulate the holiday effect.

Further subgroup analyses were performed by age (<65 vs. ≥65 years) and gender (male vs. female) to identify the underlying vulnerable population. Moreover, the relationship between air pollution and CHD hospitalization throughout the hot season (April–September) and the cold season (October–March) was investigated.

All the statistical analyses and visualization, including “DLNM” and “Spline” packages, were performed using R software version 3.6.1 (http://www.R-project.org). *P* < 0.05 (bilateral) was considered statistically significant in statistical tests.

### 2.5. Sensitivity analysis

Based on the original single-pollutant model, we considered coexisting pollutants and built the two-pollutant model to evaluate the stability of the single-pollutant model developed in this study. In addition, to test the robustness of the model, sensitivity analysis was conducted by changing the dfs in the ns functions of air pollutants (3–5 dfs), meteorological variables (3–5 dfs), and time (6–8 dfs per year).

## 3. Results

### 3.1. Descriptive analysis

This study included 16,656 CHD cases from 1 January 2014 to 31 December 2021. A majority of the cases were male patients, at 9,967 (against 6,689 female patients), and people aged ≥65 years, at 11,190 (against 5,466 people aged <65 years). In addition, the proportion of CHD cases in the hot and cold seasons was 50.49 and 49.51%, respectively. The basic characteristics of patients with CHD and the data for meteorological factors and environmental pollutants are shown in Table 1.

**Table 1:** Summary statistics of air pollutants, meteorological factors, and outpatients for CHD in Hefei, 2014–2021.

**Variable**	**Number**	**Mean (SD)**	**Centiles**					
			**Min**	**Median**	**IQR**	**Max**	**P10**	**P90**
Admissions	16,656	5.7 (3.2)	0	5	5	22	2	10
Male	9,967	3.4 (2.3)	0	3	3	13	1	6
Female	6,689	2.3 (1.7)	0	2	2	11	0	5
Age < 65	5,466	1.9 (1.6)	0	2	2	10	0	4
Age ≥ 65	11,190	3.8 (2.4)	0	3	3	16	1	7
Hot season	8,409	2.8 (3.6)	0	0	5	21	0	8
Cold season	8,247	2.9 (3.7)	0	0	5	22	0	8
Mean temperature, °C	–	16.7 (9.1)	−6.2	17.3	15.8	34.8	3.9	28.0
Relative humidity, %	–	76.9 (12.4)	32	78	17	99	60	93
PM_2.5_, μg/m^3^	–	52.0 (36.1)	0	43	40	353	18	97
PM_10_, μg/m^3^	–	79.2 (44.1)	0	72	54	417	32	132
NO_2_, μg/m^3^	–	39.0 (18.1)	8	35	23	137	21	64
SO_2_, μg/m^3^	–	11.5 (7.7)	2	9	9	63	4	22
CO, μg/m^3^	–	0.9 (0.3)	0.3	0.8	0.4	2.8	0.5	1.3
O_3_, μg/m^3^	–	85.4 (43.7)	4	77	63	269	35	148

PM_2.5_, particulate matter ≤ 2.5 μm in aerodynamic diameter; PM_10_, particulate matter ≤ 10 μm in aerodynamic diameter; NO_2_, nitrogen dioxide; CO, carbon monoxide; SO_2_, sulfur dioxide; O_3_, ozone; SD, standard deviation; IQR, interquartile range; CHD, coronary heart disease.

**Figure 2:**
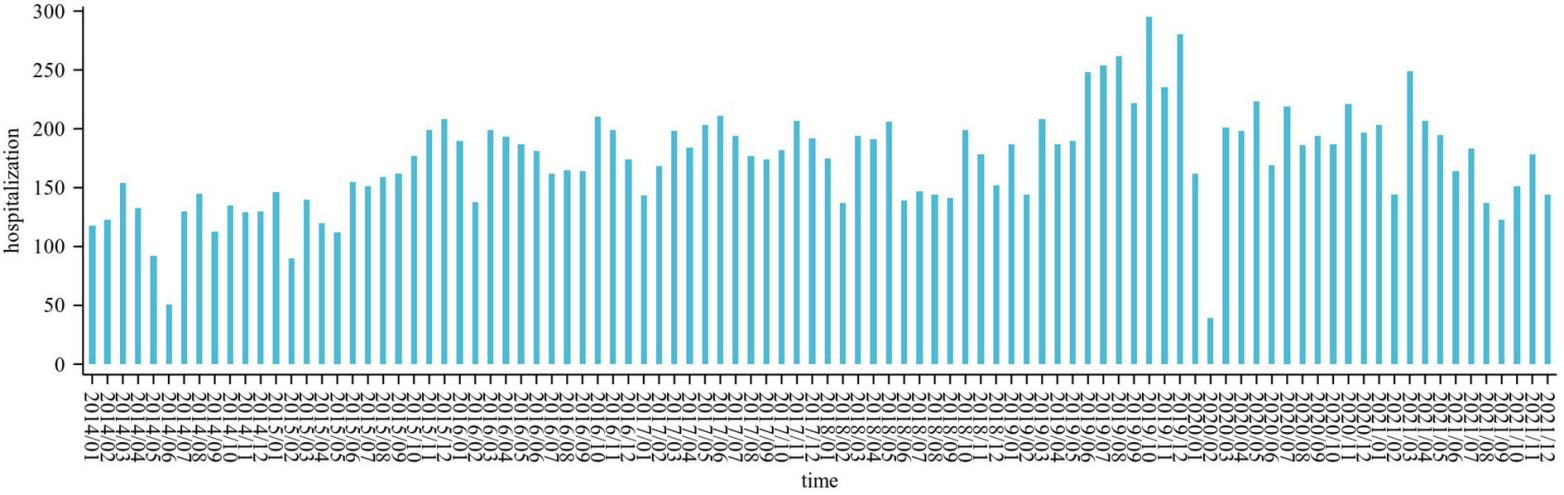
Barplot showing the temporal trends of CHD hospitalization.

**Figure 3:**
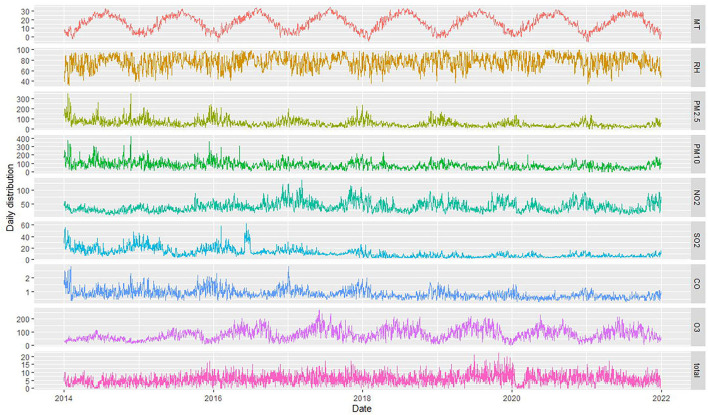
Time series of air pollutants, meteorological factors, and CHD patient hospitalizations in Hefei from 2014 to 2021 (MT represents mean temperature, RH represents relative humidity, and total represents the total number of CHD hospitalization).

The daily average concentration of NO_2_ was 39.0 μg/m^3^ (8–137 μg/m^3^), PM_2.5_ was 52.0 μg/m^3^ (0–353 μg/m^3^), SO_2_ was 11.5 μg/m^3^ (2–63 μg/m^3^), O_3_ was 85.4 μg/m^3^ (4–269 μg/m^3^), and CO was 0.9 μg/m^3^ (0.3–2.8 ug/m^3^). The number of CHD hospitalizations per day, during the study, ranged from 0 to 22 (Figure 2). The time series distribution of air pollutants in Hefei is displayed in Figure 3, whereas, the Spearman rank correlation analysis and scatter diagram are shown in Supplementary Figure S1.

### 3.2. The association between air pollution exposure and CHD hospitalization

**Figure 4:**
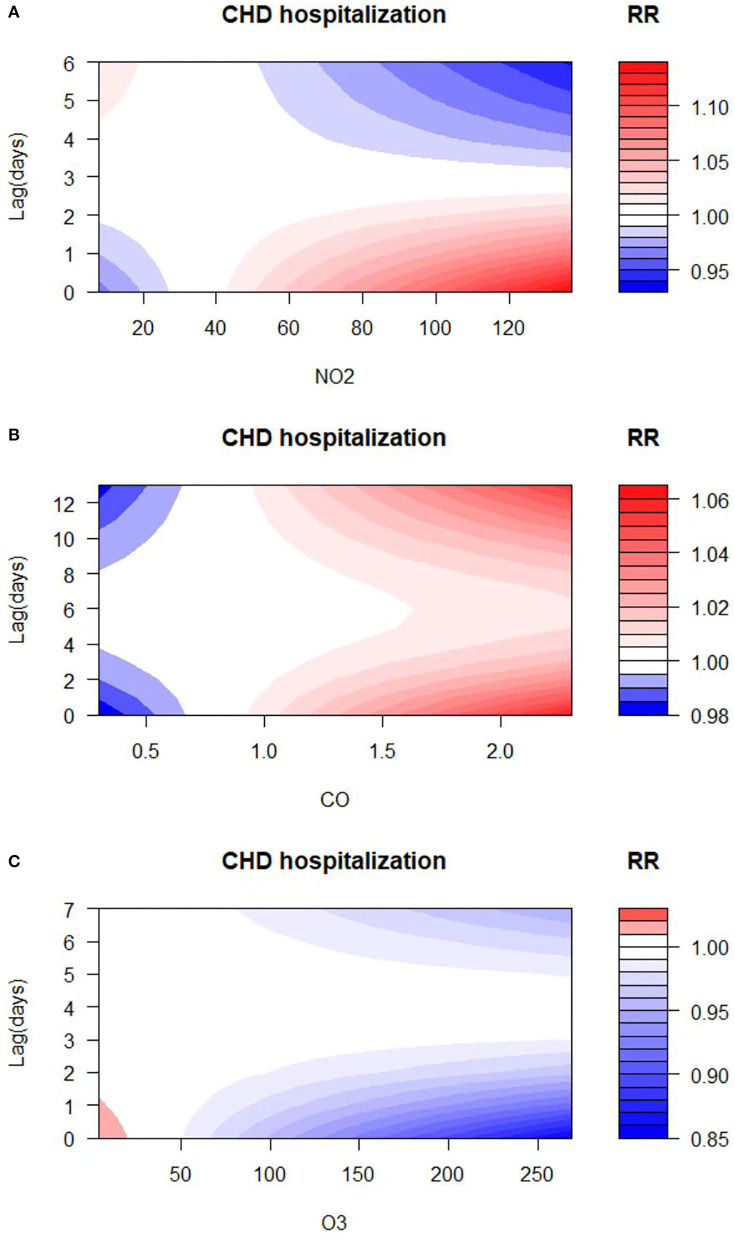
Relative risk (RR) contour plots for CHD hospitalization during the lag periods of NO_2_ **(A)**, CO **(B)**, and O_3_ **(C)** in Hefei, China, 2014–2021.

The exposure–response relationships between exposure to air pollutants (NO_2_, CO, and O_3_) with different lag days and CHD admissions are shown in Figures 4A–C. The high concentrations of NO_2_ and CO exposures showed a strong correlation with CHD hospitalization (reference concentration 35 and 0.8 μg/m^3^, respectively). On the other hand, the elevation of O_3_ levels revealed a negative correlation with CHD hospitalization (reference concentration 77 μg/m^3^). The concentration–response relationship among NO_2_, CO, O_3_, and CHD admissions is shown in Supplementary Figure S2. Accordingly, to illustrate the association between air pollutant exposure and the rate of CHD hospitalization, we analyzed the specific lag effect of the rate of CHD hospitalization.

### 3.3. The association between NO_2_ exposure and CHD hospitalization

**Figure 5:**
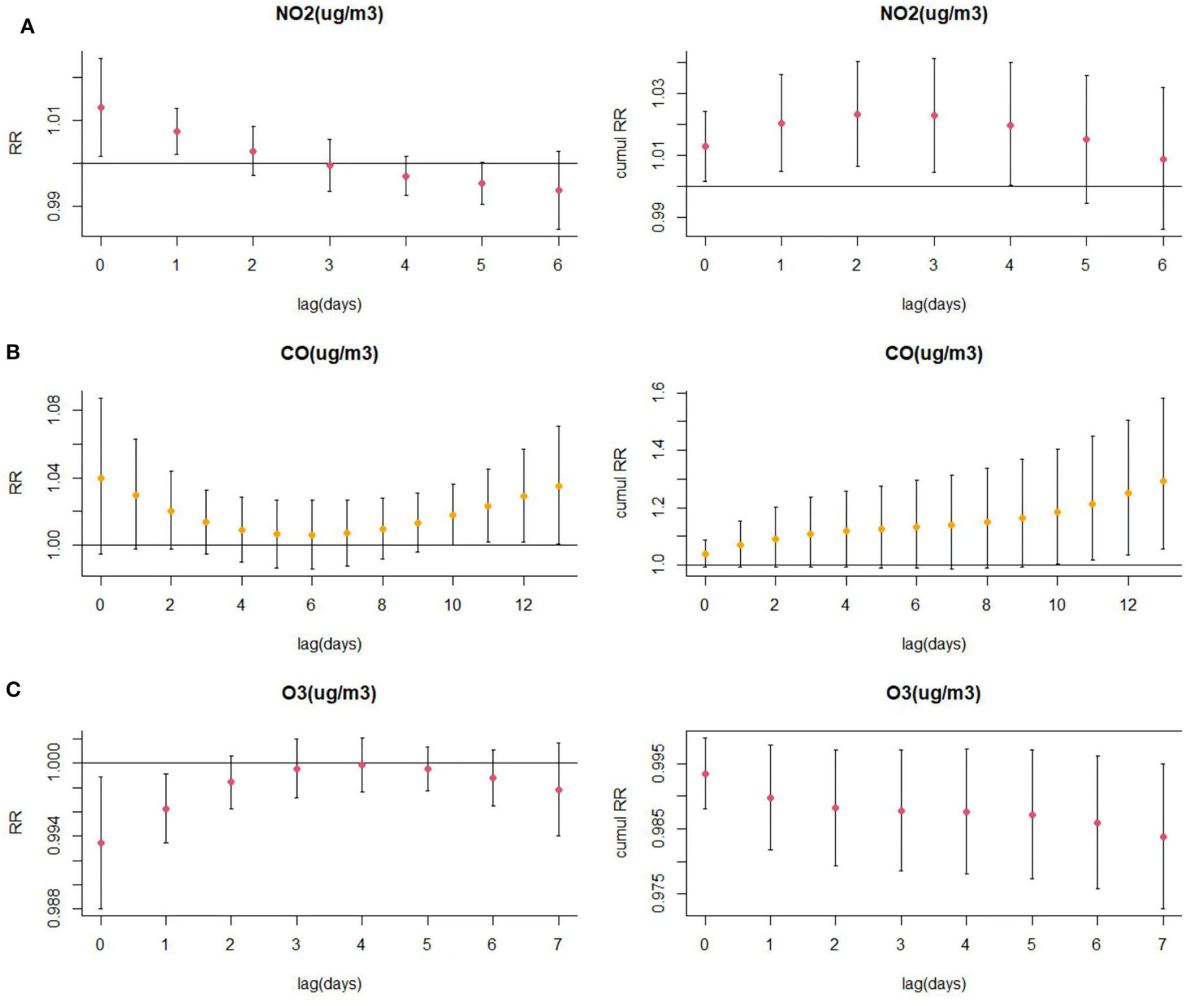
**(A)** Lagged specific relative risk (%) and **(B, C)** cumulative risk (%) of CHD hospitalization for each 10 (or 1) unit increase in daily average air pollution concentration in the model.

**Figure 6:**
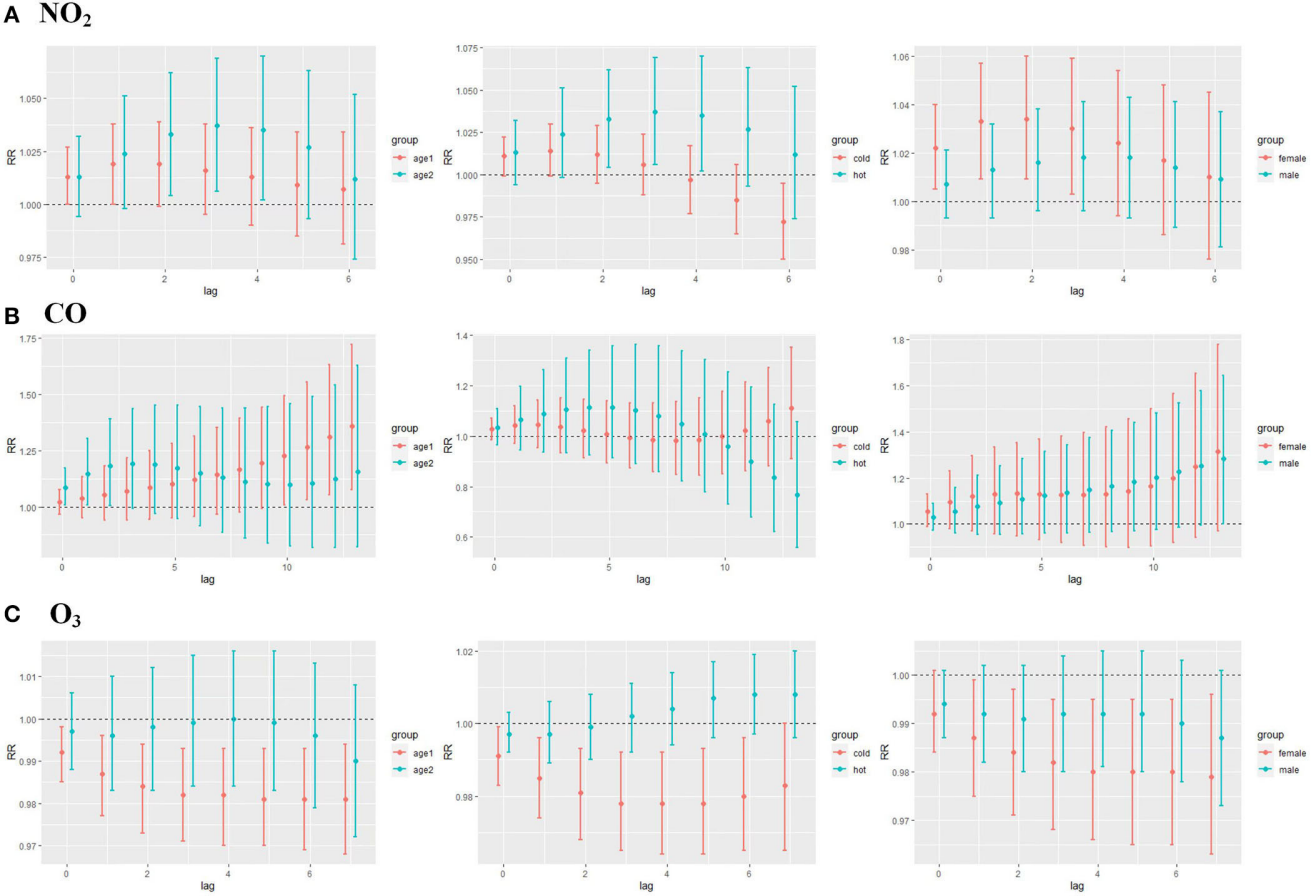
**(A)** CHD lag-specific relative risks (95% CI) per 10 unit increase in the daily concentrations of NO_2_ in models stratified by age, gender, and season; **(B)** CHD lag-specific relative risks (95% CI) per 1 unit increase in the daily concentrations of CO in models stratified by age, gender, and season; **(C)** CHD lag-specific relative risks (95% CI) per 10 unit increase in the daily concentrations of O_3_ in models stratified by age, gender, and season (age1 refers to people ≥65 years of age and age2 refers to people < 65 years of age, cold season is from October to March, and the hot season is from April to September).

Results of the association between NO_2_ exposure and CHD hospitalization revealed a strong correlation between NO_2_ and the rate of CHD hospitalization in the single-pollutant model (lag0, RR: 1.013, 95%CI: 1.002–1.024; and lag1, RR: 1.007, 95%CI: 1.002–1.013, per 10 μg/m^3^ increase in NO_2_ concentration) (Figure 5A). The results of the exposure–response relationship and the lag–response relationship of NO_2_ are shown in Supplementary Table S1. Subgroup analyses showed that the association between NO_2_ exposure and the rate of CHD hospitalization increased in female patients (RR: 1.022, 95%CI: 1.005–1.040, lag0) and patients aged < 65 years (RR: 1.037, 95% CI: 1.006–1.069, lag3), while there was no statistical significance between NO_2_ exposure and the rate of CHD hospitalization among male patients (RR: 1.007, 95% CI: 0.993–1.021, lag0) and patients aged ≥65 years (RR: 1.016, 95% CI: 0.995–1.038, lag3). Moreover, exposure to NO_2_ during hot seasons was related to an increased rate of CHD hospitalization (RR: 1.037, 95%CI: 1.006–1.069, lag3). Interestingly, the relationship between exposure to NO_2_ during hot seasons and increased rate of CHD hospitalization was not statistically significant in the cold season (RR: 1.006, 95% CI: 0.988–1.024, lag3) (Figure 6A; Supplementary Table S2).

### 3.4. The association between CO exposure and CHD hospitalization

As displayed in Figure 5B, there was a strong correlation between CO and CHD hospitalization rate in the single-pollutant model (lag13, RR: 1.035, 95%CI: 1.001–1.071, per 1 μg/m^3^ increase in CO concentration). We further explored the exposure–response relationship and the lag–response relationship of CO (Supplementary Table S3). Subgroup analyses indicated that CO exposure positively correlated with the rate of CHD hospitalization among patients aged above 65 years (RR: 1.360, 95%CI: 1.075–1.722, lag13). Yet, we found no association among CO exposure and other subgroups such as male patients (RR: 1.283, 95%CI: 1.000–1.246, lag13), female patients (RR: 1.315, 95%CI: 0.971–1.780, lag13), people < 65 years of age (RR: 1.156, 95%CI: 0.822–1.627, lag13), cold seasons (RR: 1.111, 95%CI: 0.911–1.353, lag13), and hot seasons (RR: 0.767, 95%CI: 0.558–1.056, lag13) (Figure 6B; Supplementary Table S4).

### 3.5. The association between O_3_ exposure and CHD hospitalization

Results of the single pollutant model showed that O_3_ correlated with the rate of CHD hospitalization (lag0, RR: 0.993, 95%CI: 0.988–0.999; and lag1, RR: 0.996, 95%CI: 0.993–0.999, per 10 μg/m^3^ increase in O_3_ concentration) (Figure 5C). We further explored the exposure–response relationship and the lag–response relationship of O_3_ and the results are presented in Supplementary Table S5. Further subgroup analyses revealed that O_3_ exposure negatively correlated with the rate of CHD hospitalization in subgroups of female patients (RR: 0.979, 95%CI: 0.963–0.996, lag7), people ≥65 years of age (RR: 0.981, 95%CI: 0.968–0.994, lag7), and during the cold season (RR: 0.978, 95%CI: 0.964–0.992, lag4). On the other hand, O_3_ exposure and the rate of CHD hospitalization among male patients (RR: 0.987, 95%CI: 0.973–1.001, lag7), people < 65 years of age (RR: 0.990, 95%CI: 0.972–1.008, lag7), and in hot seasons (RR: 1.004, 95%CI: 0.994–1.014, lag7) were not statistically significant (Figure 6C; Supplementary Table S6).

### 3.6. Sensitivity analysis

Considering that various pollutants may have collinearity with each other when a single pollutant model is used to assess the effect of a pollutant on the quantity of CHD hospitalization, another pollutant was included as an independent variable to explore the association between other coexisting pollutants and CHD hospitalization. The results of fitting the two-pollutant model showed that the results were unchanged (Tables 2–4). We also evaluated the stability of the model by varying degrees of freedom (4–6 dfs/year) and meteorological factors (4–6 dfs). The results demonstrated that the model fits well and produced reliable results (Supplementary Figures S3–S5).

**Table 2:** The association between an increase of 10 μg/m^3^ of NO_2_ and CHD admission in Hefei, 2014–2021 (dual pollutant model).

**Lag**	**NO_2_ + PM_2.5_**	**NO_2_ + PM_10_**	**NO_2_ + O_3_**	**NO_2_ + SO_2_**	**NO_2_ + CO**
0	1.015 (1.003–1.027)^*^	1.018 (1.005–1.031)^*^	1.013 (1.002–1.025)^*^	1.022 (1.009–1.034)*	1.012 (0.999–1.025)^*^
1	1.008 (1.003–1.014)^*^	1.010 (1.004–1.016)^*^	1.008 (1.002–1.013)^*^	1.011 (1.005–1.017)^*^	1.007 (1.001–1.013)^*^
2	1.003 (0.997–1.009)	1.003 (0.998–1.009)	1.003 (0.997–1.009)	1.004 (0.998–1.009)	1.003 (0.997–1.009)
3	0.999 (0.993–1.005)	0.999 (0.993–1.005)	1.000 (0.994–1.006)	0.999 (0.993–1.005)	1.000 (0.994–1.006)
4	0.997 (0.992–1.002)	0.997 (0.992–1.001)	0.997 (0.993–1.002)	0.996 (0.991–1.001)	0.997 (0.993–1.002)
5	0.995 (0.990–1.000)	0.995 (0.991–1.000)	0.995 (0.990–1.000)	0.995 (0.990–1.000)	0.995 (0.990–1.000)
6	0.994 (0.985–1.003)	0.995 (0.985–1.004)	0.994 (0.985–1.003)	0.994 (0.985–1.003)	0.994 (0.985–1.003)
0–0	1.015 (1.003–1.027)^*^	1.018 (1.005–1.031)^*^	1.013 (1.002–1.025)^*^	1.022 (1.009–1.034)^*^	1.012 (0.999–1.025)
0–1	1.024 (1.006–1.041)^*^	1.028 (1.010–1.046)^*^	1.021 (1.005–1.037)^*^	1.033 (1.015–1.052)^*^	1.019 (1.001–1.038)^*^
0–2	1.027 (1.008–1.046)^*^	1.031 (1.012–1.051)^*^	1.024 (1.007–1.041)^*^	1.037 (1.018–1.057)^*^	1.022 (1.002–1.042)^*^
0–3	1.026 (1.007–1.046)^*^	1.030 (1.010–1.051)^*^	1.024 (1.005–1.043)^*^	1.036 (1.015–1.056)^*^	1.022 (1.001–1.043)^*^
0–4	1.023 (1.002–1.044)^*^	1.027 (1.006–1.049)^*^	1.021 (1.001–1.041)^*^	1.032 (1.010–1.053)^*^	1.019 (0.997–1.041)
0–5	1.018 (0.997–1.040)	1.022 (1.000–1.045)	1.016 (0.995–1.037)	1.026 (1.004–1.049)^*^	1.014 (0.992–1.037)
0–6	1.012 (0.988–1.036)	1.017 (0.992–1.042)	1.009 (0.987–1.033)	1.020 (0.996–1.045)	1.008 (0.984–1.033)

* P < 0.0.5.

CHD, coronary heart disease; PM_2.5_, particulate matter < 2.5 μm in aerodynamic diameter; PM_10_, particulate matter < 10 μm in aerodynamic diameter; SO_2_, sulfur dioxide; NO_2_, nitrogen dioxide; O_3_, ozone; CO, carbon monoxide.

**Table 3:** The association between an increase of 1ug/m3 of CO and CHD admission in Hefei, 2014–2021 (dual pollutant model).

**Lag**	**CO + PM_2.5_**	**CO + PM_10_**	**CO + O_3_**	**CO + SO_2_**	**CO + NO_2_**
0	1.057 (1.002–1.116)^*^	1.054 (1.002–1.108)^*^	1.045 (0.999–1.094)	1.049 (1.001–1.099)^*^	1.011 (0.963–1.060)
1	1.041 (1.002–1.082)^*^	1.039 (1.002–1.076)^*^	1.034 (1.001–1.068)^*^	1.036 (1.002–1.071)^*^	1.010 (0.977–1.045)
2	1.027 (1.001–1.054)^*^	1.026 (1.001–1.051)^*^	1.024 (1.001–1.048)^*^	1.024 (1.000–1.048)	1.010 (0.986–1.034)
3	1.017 (0.997–1.037)	1.016 (0.996–1.036)	1.016 (0.997–1.036)	1.015 (0.996–1.035)	1.010 (0.991–1.030)
4	1.009 (0.990–1.029)	1.009 (0.990–1.029)	1.011 (0.992–1.031)	1.009 (0.990–1.029)	1.011 (0.991–1.030)
5	1.005 (0.985–1.026)	1.006 (0.985–1.026)	1.008 (0.988–1.029)	1.006 (0.986–1.026)	1.011 (0.991–1.032)
6	1.003 (0.983–1.025)	1.004 (0.984–1.025)	1.007 (0.987–1.028)	1.005 (0.985–1.025)	1.013 (0.992–1.034)
7	1.004 (0.984–1.025)	1.005 (0.985–1.025)	1.008 (0.989–1.028)	1.006 (0.986–1.026)	1.014 (0.994–1.034)
8	1.007 (0.988–1.026)	1.008 (0.989–1.026)	1.010 (0.992–1.029)	1.008 (0.990–1.027)	1.016 (0.997–1.034)
9	1.011 (0.994–1.029)	1.012 (0.994–1.029)	1.014 (0.997–1.031)	1.012 (0.995–1.030)	1.017 (1.000–1.035)
10	1.017 (0.999–1.035)	1.017 (0.999–1.036)	1.018 (1.000–1.037)	1.018 (0.999–1.036)	1.019 (1.001–1.038)^*^
11	1.024 (1.002–1.046)^*^	1.024 (1.002–1.046)^*^	1.024 (1.002–1.046)^*^	1.024 (1.002–1.046)^*^	1.021 (1.000–1.043)
12	1.031 (1.004–1.059)^*^	1.031 (1.003–1.059)^*^	1.029 (1.002–1.057)^*^	1.030 (1.003–1.058)^*^	1.024 (0.996–1.052)
13	1.039 (1.004–1.075)^*^	1.038 (1.003–1.074)^*^	1.035 (1.001–1.071)^*^	1.037 (1.003–1.073)^*^	1.026 (0.991–1.061)
0–0	1.057 (1.002–1.116)^*^	1.054 (1.002–1.108)^*^	1.045 (0.999–1.094)^*^	1.049 (1.001–1.099)^*^	1.011 (0.963–1.060)
0–1	1.101 (1.004–1.206)^*^	1.094 (1.005–1.192)^*^	1.081 (1.001–1.167)^*^	1.087 (1.004–1.177)^*^	1.021 (0.941–1.107)
0–2	1.131 (1.007–1.269)^*^	1.123 (1.008–1.251)^*^	1.107 (1.003–1.220)^*^	1.113 (1.006–1.231)^*^	1.031 (0.930–1.143)
0–3	1.149 (1.010–1.308)^*^	1.141 (1.010–1.288)^*^	1.124 (1.006–1.257)^*^	1.130 (1.008–1.267)^*^	1.041 (0.927–1.169)
0–4	1.160 (1.011–1.331)^*^	1.151 (1.011–1.312)^*^	1.137 (1.008–1.283)^*^	1.140 (1.008–1.290)^*^	1.053 (0.929–1.192)
0–5	1.166 (1.010–1.345)^*^	1.158 (1.010–1.327)^*^	1.146 (1.008–1.304)^*^	1.147 (1.007–1.307)^*^	1.065 (0.933–1.214)
0–6	1.170 (1.009–1.356)^*^	1.163 (1.008–1.341)^*^	1.154 (1.007–1.323)^*^	1.152 (1.004–1.323)^*^	1.078 (0.939–1.238)
0–7	1.175 (1.008–1.369)^*^	1.168 (1.006–1.356)^*^	1.164 (1.007–1.345)^*^	1.159 (1.002–1.340)^*^	1.093 (0.945–1.264)
0–8	1.183 (1.008–1.387)^*^	1.177 (1.007–1.376)^*^	1.176 (1.009–1.370)^*^	1.169 (1.003–1.362)^*^	1.110 (0.952–1.294)
0–9	1.196 (1.013–1.412)^*^	1.191 (1.011–1.402)^*^	1.192 (1.015–1.400)^*^	1.183 (1.007–1.390)^*^	1.129 (0.961–1.326)
0–10	1.216 (1.022–1.447)^*^	1.211 (1.021–1.437)^*^	1.214 (1.025–1.437)^*^	1.204 (1.017–1.425)^*^	1.151 (0.972–1.363)
0–11	1.245 (1.038–1.493)^*^	1.240 (1.036–1.484)^*^	1.242 (1.040–1.483)^*^	1.232 (1.032–1.471)^*^	1.176 (0.985–1.404)
0–12	1.284 (1.058–1.557)^*^	1.278 (1.056–1.546)^*^	1.279 (1.060–1.543)^*^	1.270 (1.052–1.532)^*^	1.203 (0.997–1.452)
0–13	1.334 (1.082–1.643)^*^	1.326 (1.079–1.630)^*^	1.324 (1.081–1.621)^*^	1.317 (1.075–1.613)^*^	1.234 (1.007–1.512)^*^

* P < 0.05.

CHD, coronary heart disease; PM_2.5_, particulate matter < 2.5 μm in aerodynamic diameter; PM_10_, particulate matter < 10 μm in aerodynamic diameter; SO_2_, sulfur dioxide; NO_2_, nitrogen dioxide; O_3_, ozone; CO, carbon monoxide.

**Table 4:** The association between an increase of 10 μg/m^3^ of O_3_ and CHD admission in Hefei, 2014–2021 (dual pollutant model).

**Lag**	**O_3_ + PM_2.5_**	**O_3_ + PM_10_**	**O_3_ + CO**	**O_3_ + SO_2_**	**O_3_ + NO_2_**
0	0.992 (0.986–0.998)^*^	0.993 (0.987–0.998)^*^	0.992 (0.987–0.998)^*^	0.993 (0.988–0.999)^*^	0.991 (0.986–0.997)^*^
1	0.996 (0.993–0.999)^*^	0.996 (0.993–0.999)^*^	0.996 (0.993–0.999)^*^	0.996 (0.993–0.999)^*^	0.995 (0.992–0.998)^*^
2	0.998 (0.996–1.000)	0.998 (0.996–1.001)	0.998 (0.996–1.001)	0.998 (0.996–1.001)	0.998 (0.996–1.000)
3	1.000 (0.997–1.002)	1.000 (0.997–1.002)	1.000 (0.997–1.002)	1.000 (0.997–1.002)	1.000 (0.997–1.002)
4	1.000 (0.998–1.002)	1.000 (0.998–1.002)	1.000 (0.998–1.002)	1.000 (0.998–1.002)	1.000 (0.998–1.003)
5	1.000 (0.998–1.002)	1.000 (0.998–1.001)	1.000 (0.998–1.002)	1.000 (0.998–1.001)	1.000 (0.998–1.002)
6	0.999 (0.997–1.001)	0.999 (0.997–1.001)	0.999 (0.997–1.001)	0.999 (0.997–1.001)	0.999 (0.997–1.001)
7	0.998 (0.994–1.002)	0.998 (0.994–1.002)	0.998 (0.994–1.001)	0.998 (0.994–1.002)	0.998 (0.994–1.002)
0–0	0.992 (0.986–0.998)^*^	0.993 (0.987–0.998)^*^	0.992 (0.987–0.998)^*^	0.993 (0.988–0.999)^*^	0.991 (0.986–0.997)^*^
0–1	0.988 (0.979–0.996)^*^	0.989 (0.980–0.997)^*^	0.988 (0.980–0.996)^*^	0.989 (0.981–0.997)^*^	0.987 (0.979–0.995)^*^
0–2	0.986 (0.976–0.995)^*^	0.987 (0.978–0.996)^*^	0.986 (0.977–0.996)^*^	0.988 (0.979–0.997)^*^	0.985 (0.976–0.994)^*^
0–3	0.985 (0.976–0.995)^*^	0.987 (0.977–0.996)^*^	0.986 (0.977–0.996)^*^	0.987 (0.978–0.997)^*^	0.985 (0.975–0.994)^*^
0–4	0.986 (0.976–0.996)^*^	0.987 (0.977–0.996)^*^	0.986 (0.976–0.996)^*^	0.987 (0.978–0.997)^*^	0.985 (0.975–0.995)^*^
0–5	0.985 (0.975–0.996)^*^	0.986 (0.976–0.996)^*^	0.986 (0.976–0.996)^*^	0.987 (0.977–0.997)^*^	0.985 (0.975–0.995)^*^
0–6	0.984 (0.974–0.995)^*^	0.985 (0.975–0.995)^*^	0.985 (0.974–0.995)^*^	0.986 (0.976–0.996)^*^	0.984 (0.974–0.994)^*^
0–7	0.982 (0.971–0.993)^*^	0.983 (0.972–0.994)^*^	0.982 (0.971–0.994)^*^	0.984 (0.973–0.995)^*^	0.982 (0.971–0.993)^*^

* P < 0.05.

CHD, coronary heart disease; PM_2.5_, particulate matter < 2.5 μm in aerodynamic diameter; PM_10_, particulate matter < 10 μm in aerodynamic diameter; SO_2_, sulfur dioxide; NO_2_, nitrogen dioxide; O_3_, ozone; CO, carbon monoxide.

## 4. Discussion

Coronary heart disease (CHD) is a major chronic disease that threatens human health and negatively affects patients' quality of life globally. In severe cases of the disease, heart failure, myocardial infarction, and other adverse consequences may occur. More than 40% of deaths in China can be attributed to CHD (24). This implies that the potential contribution of air pollutants to the burden of CHD is significant in China. Nonetheless, the exact mechanism of air pollutants and CV systems remains inconclusive and inadequate. Previously, studies have reported the potential for air pollutants to affect the CV system directly or indirectly. The action mechanisms of air pollution may include epigenetic changes, inflammatory reactions, and other mechanisms, which can stimulate a series of pathological processes such as endothelial damage, vascular dysfunction, autonomic and neuroendocrine dysfunction, thrombosis, and atherosclerosis (25, 26). Exposure to air pollutants may worsen heart failure and reduce heart rate variability in the elderly due to decreased circulatory function and immunity (27).

With the accelerating process of industrialization and urbanization, air pollution has become a big threat to people's health, particularly in densely populated cities. In this study, we investigated the association between air pollutants and CHD hospitalization. The study's findings indicated that exposure to high concentrations of NO_2_ and CO correlated with an increased rate of CHD hospitalization. Furthermore, the findings revealed that exposure to O_3_ correlated with a lower rate of CHD hospitalization. Sensitivity analysis demonstrated that the model suited well and the outcomes were robust.

The current research found that exposure to NO_2_ increases the rate of CHD hospitalization. These findings are supported by previous epidemiological results (28–30). The NO_2_ is an index of traffic mixtures, such as ultrafine particles and diesel exhaust black carbon. Previously, studies involving experimental models have demonstrated the potential of diesel exhaust and ultrafine particles to cause noxious biological reactions (31). Thus, NO_2_ may promote the development of CHD *via* mechanisms such as the induction of inflammation and oxidative stress, coronary endothelial vasoconstriction, abnormal regulation of the cardiac autonomic nervous system, increased blood viscosity, and vasospasm. These may eventually contribute to plaque rupture, ischemia, and arrhythmias in CHD hospitalization (32, 33).

The present study revealed that O_3_ was negatively correlated with the rate of CHD hospitalization, which may be related to the newly discovered effects of O_3_. Studies have shown that O_3_ has effective antibacterial and antioxidant defense abilities (34) and is also related to the regulation of immune responses and accelerated wound healing (35, 36). Animal research has shown that O_3_ exposure can increase serum concentrations of cholesterol, triglycerides, and low-density (LDL) and very low-density (VLDL) lipoproteins. The risk of CHD increases with an increase in levels of lipid and lipoprotein concentrations in the human body, which is contrary to our findings. It is important to note that O_3_ is very active chemically and it can react with other air pollutants to form new compounds (37), making it unstable in the environment. Accordingly, we should be cautious in explaining the effects of O_3_ exposure. The mechanism of O_3_ in CHD remains to be further studied.

We revealed that CO exposure was related to an elevated rate of CHD hospitalization, which is consistent with previous epidemiological findings (38). Inhaled CO can combine with hemoglobin and form carboxyhemoglobin, thereby reducing the oxygen-carrying capacity of hemoglobin and leading to cellular hypoxia (39). Several possible biological mechanisms have been proposed that underlie the associations between low ambient CO concentrations and CVD. They include cardiac dysfunction (40), systemic inflammation (41), oxidative stress, and thrombotic reactions (42). Both animal models and population-based cohort studies confirm the cardiotoxicity of CO (40, 41). It has been proven that the cardiotoxicity of CO is caused by the dual effects of tissue hypoxia and direct effects on the myocardium. Chen et al. (42) carried out longitudinal research where they investigated 61 patients with CVD. The authors observed that the high-sensitivity c-reactive protein was elevated with short-term exposure to CO. They further reported that Fibrinogen and D-dimer were increased in response to environmental CO exposure. These biomarkers have been associated with thrombotic responses (43). Overall, the above evidence and our outcomes support a correlation between environmental CO exposure and CHD hospitalization risk.

To elucidate the correlation between the four aforementioned air pollutants and the rate of CHD hospitalization, further subgroup analyses regarding varied age, sex, and season groups were conducted. The results showed a correlation between NO_2_ exposure and an elevated CHD hospitalization rate in young, female patients with CHD during the hot season. This may be because NO_2_ pollution mainly comes from car exhaust. Young people can stay in such an environment longer than the elderly because they engage in more outdoor activities. Women's immunity is weaker than that of men, and the reason that the effect is greater in hot seasons may be that the rise in temperature accelerates the spread of NO_2_ in the air and reacts with other pollutants. However, the association between O_3_ and CHD hospitalization was more obvious in female patients, patients aged over 65 years, and during the cold season. These findings may be attributed to the fact that women and the elderly are more sensitive to the external environment. Moreover, the photoreaction rate is relatively high in the summer, and the second-generation concentration of O_3_ is higher than that in the winter. In terms of CO, it was revealed that CO exposure and CHD hospitalization was more common among patients aged above 65 years. This result implies that age-related serious brain systemic inflammatory environment and lower cerebrovascular hemodynamics might cause CHD cases among the elderly who are more sensitive to the mediators of CO.

Noteworthy, the current study is not without shortcomings. First, we did not consider the spatial heterogeneity of urban air pollution and we calculated the average value of 10 fixed sites by considering the exposure index of NO_2_, O_3_, and CO. Anyway, almost all the families of hospitalized patients with CHD are within the detection range of urban monitoring stations, which avoids significant spatial differences to some extent. Second, the time-series study methodology is naturally an ecological design, which may be vulnerable to ecological fallacies. Third, the lack of detailed individual information for patients with CHD, such as exercise, mental and mental stress, smoking, and heavy drinking, may have limited subgroup analyses. Fourth, we, unfortunately, failed to obtain specific individual diagnostic data to distinguish between first CHD outpatient visits and recurrent CHD outpatient visits, thus restricting the potential clinical impact of the study results. Finally, this is a single city study. Because of geographical and climatic divergence, the results need to be further verified when extrapolated to other regions.

Despite the shortcomings, our study also has some advantages. Since previous studies have focused more on the health effects of particulate matter on CHD, we have investigated the meteorological factors of NO_2_, CO, and O_3_ on the risk of CHD hospitalization. In addition, we have identified that the correlation between air pollution exposure and hospitalization risk of CHD may be influenced by season, gender, and age. This information may form a basis upon which healthcare practitioners may formulate novel protective measures to minimize the adverse health effects of air pollution on CHD risk earlier.

## 5. Conclusion

Overall, this time-series study shows that short-term exposures to NO_2_ and CO positively correlate with the risk of CHD hospitalization. However, O_3_ exposure is related to a decreased risk of CHD. Pollutant effects are different in different populations, according to stratified analysis. Consequently, to effectively reduce the risk of CHD admission, it is essential to construct a sound environmental monitoring and feedback system. For susceptible populations, appropriate preventive measures should be developed to avoid unnecessary and harmful exposure.

## Data availability statement

The original contributions presented in the study are included in the article/Supplementary material, further inquiries can be directed to the corresponding authors.

## Author contributions

Y-TF: conceptualization, methodology, and software. C-FL: data curation and writing—original draft preparation. MH, YF, Y-SH, and YC: writing—reviewing and editing. CC: visualization and investigation. YF, R-DZ, and XF: supervision. L-QJ: software and validation. All authors contributed to the article and approved the submitted version.
