# Supplementary material for: Association between air pollution exposure and coronary heart disease hospitalization in a humid sub-tropical region of China: A time-series study
Source: Front Public Health. 2023 Jan 11;10:1090443. doi: 10.3389/fpubh.2022.1090443 (PMC9874291; doi:10.3389/fpubh.2022.1090443)

Table S1 The association between an increase of 10ug/m^3^ of NO_2_ and CHD admission in Hefei, 2014-2021

| Single-day | Relative risk (95%,confidence interval) | Multi-day | Relative risk (95%, confidence interval) |
| --- | --- | --- | --- |
| 0 | 1.013(1.002-1.024) * | 0-0 | 1.013(1.002-1.024) * |
| 1 | 1.007(1.002-1.013) * | 0-1 | 1.020(1.005-1.036) * |
| 2 | 1.003(0.997-1.009) | 0-2 | 1.023(1.007-1.040) * |
| 3 | 1.000(0.994-1.006) | 0-3 | 1.023(1.005-1.041) * |
| 4 | 0.997(0.993-1.002) | 0-4 | 1.020(1.000-1.040) |
| 5 | 0.995(0.990-1.000) | 0-5 | 1.015(0.995-1.036) |
| 6 | 0.994(0.985-1.003) | 0-6 | 1.009(0.986-1.032) |

*: *P < 0.05*

Abbreviations: CHD: coronary heart disease; NO_2_: nitrogen dioxide.

Table S2 The association between an increase of 10ug/m^3^ of NO_2_ and CHD admission in different subgroups (relative risk and 95% confidence interval).

| lag | male | female | age<65yeras | age≥65years | | hot season | cold season |
| --- | --- | --- | --- | --- | --- | --- | --- |
| 0 | 1.007(0.993-1.021) | 1.022(1.005-1.040) * | 1.013(0.994-1.032) | | 1.013(1.000-1.027) | 1.013(0.994-1.032) | 1.011(0.999-1.022) |
| 1 | 1.006(0.999-1.012) | 1.010(1.002-1.018) * | 1.011(1.002-1.020) * | | 1.006(0.999-1.012) | 1.011(1.002-1.020) * | 1.004(0.998-1.009) |
| 2 | 1.004(0.997-1.011) | 1.001(0.993-1.010) | 1.008(0.999-1.018) | | 1.000(0.993-1.007) | 1.008(0.999-1.018) | 0.998(0.992-1.004) |
| 3 | 1.002(0.994-1.009) | 0.996(0.987-1.005) | 1.004(0.994-1.014) | | 0.997(0.990-1.005) | 1.004(0.994-1.014) | 0.994(0.988-1.000) |
| 4 | 0.999(0.994-1.005) | 0.994(0.987-1.001) | 0.998(0.991-1.006) | | 0.996(0.991-1.002) | 0.998(0.991-1.006) | 0.991(0.986-0.995) |
| 5 | 0.997(0.991-1.003) | 0.993(0.986-1.000) | 0.992(0.984-1.000) | | 0.997(0.991-1.003) | 0.992(0.984-1.000) | 0.989(0.984-0.993) |
| 6 | 0.994(0.983-1.006) | 0.993(0.980-1.007) | 0.985(0.970-1.001) | | 0.998(0.987-1.009) | 0.985(0.970-1.001) | 0.987(0.978-0.996) |
| 0-0 | 1.007(0.993-1.021) | 1.022(1.005-1.040) * | 1.013(0.994-1.032) | | 1.013(1.000-1.027) | 1.013(0.994-1.032) | 1.011(0.999-1.022) |
| 0-1 | 1.013(0.993-1.032) | 1.033(1.009-1.057) * | 1.024(0.998-1.051) | | 1.019(1.000-1.038) | 1.024(0.998-1.051) | 1.014(0.999-1.030) |
| 0-2 | 1.016(0.996-1.038) | 1.034(1.009-1.060) * | 1.033(1.004-1.062) * | | 1.019(0.999-1.039) | 1.033(1.004-1.062) * | 1.012(0.995-1.029) |
| 0-3 | 1.018(0.996-1.041) | 1.030(1.003-1.059) * | 1.037(1.006-1.069) * | | 1.016(0.995-1.038) | 1.037(1.006-1.069) * | 1.006(0.988-1.024) |
| 0-4 | 1.018(0.993-1.043) | 1.024(0.994-1.054) | 1.035(1.002-1.070) * | | 1.013(0.990-1.036) | 1.035(1.002-1.070) * | 0.997(0.977-1.017) |
| 0-5 | 1.014(0.989-1.041) | 1.017(0.986-1.048) | 1.027(0.993-1.063) | | 1.009(0.985-1.034) | 1.027(0.993-1.063) | 0.985(0.965-1.006) |
| 0-6 | 1.009(0.981-1.037) | 1.010(0.976-1.045) | 1.012 (0.974-1.052) | | 1.007(0.981-1.034) | 1.012(0.974-1.052) | 0.972(0.950-0.995) |

*: *P < 0.05*

Abbreviations: CHD: coronary heart disease; NO_2_: nitrogen dioxide.

Table S3 The association between an increase of 1ug/m^3^ of CO and CHD admission in Hefei, 2014-2021

| Single-day | Relative risk (95%,confidence interval) | Multi-day | Relative risk (95%, confidence interval) |
| --- | --- | --- | --- |
| 0 | 1.040(0.995-1.087) | 0-0 | 1.040(0.995-1.087) |
| 1 | 1.030(0.997-1.063) | 0-1 | 1.071(0.993-1.156) |
| 2 | 1.020(0.998-1.044) | 0-2 | 1.093(0.992-1.236) |
| 3 | 1.014(0.994-1.033) | 0-3 | 1.107(0.992-1.236) |
| 4 | 1.009(0.990-1.028) | 0-4 | 1.117(0.992-1.259) |
| 5 | 1.007(0.987-1.027) | 0-5 | 1.125(0.991-1.277) |
| 6 | 1.006(0.986-1.027) | 0-6 | 1.131(0.989-1.295) |
| 7 | 1.007(0.988-1.027) | 0-7 | 1.139(0.988-1.315) |
| 8 | 1.010(0.992-1.028) | 0-8 | 1.150(0.989-1.339) |
| 9 | 1.013(0.996-1.031) | 0-9 | 1.166(0.994-1.367) |
| 10 | 1.018(1.000-1.036) | 0-10 | 1.186(1.003-1.403) * |
| 11 | 1.023(1.002-1.045) * | 0-11 | 1.214(1.018-1.448) * |
| 12 | 1.029(1.002-1.057) * | 0-12 | 1.249(1.037-1.505) * |
| 13 | 1.035(1.001-1.071) * | 0-13 | 1.293(1.058-1.581) * |

*: *P < 0.05*

Abbreviations: CHD: coronary heart disease; CO: carbon monoxide.

Table S4 The association between an increase of 1ug/m^3^ of CO and CHD admission in different subgroups (relative risk and 95% confidence interval)

| lag | male | female | age<65yeras | age≥65years | hot season | cold season |
| --- | --- | --- | --- | --- | --- | --- |
| 0 | 1.030(0.974-1.088) | 1.056(0.988-1.129) | 1.086(1.007-1.171) * | 1.020(0.968-1.075) | 1.034(0.965-1.109) | 1.028(0.986-1.072) |
| 1 | 1.024(0.985-1.066) | 1.038(0.989-1.089) | 1.056(1.001-1.114) * | 1.018(0.980-1.056) | 1.029(0.979-1.080) | 1.014(0.984-1.045) |
| 2 | 1.020(0.992-1.049) | 1.022(0.987-1.057) | 1.030(0.991-1.070) | 1.016(0.989-1.043) | 1.023(0.987-1.060) | 1.001(0.980-1.023) |
| 3 | 1.016(0.993-1.041) | 1.009(0.981-1.039) | 1.010(0.978-1.043) | 1.015(0.993-1.038) | 1.016(0.984-1.048) | 0.992(0.975-1.011) |
| 4 | 1.014(0.990-1.038) | 1.002(0.973-1.031) | 0.996(0.964-1.029) | 1.015(0.992-1.038) | 1.008(0.976-1.041) | 0.987(0.970-1.005) |
| 5 | 1.013(0.988-1.038) | 0.998(0.968-1.028) | 0.987(0.954-1.021) | 1.016(0.992-1.040) | 0.999(0.966-1.034) | 0.986(0.968-1.004) |
| 6 | 1.012(0.987-1.038) | 0.997(0.967-1.028) | 0.982(0.949-1.017) | 1.017(0.993-1.041) | 0.990(0.957-1.025) | 0.987(0.969-1.005) |
| 7 | 1.012(0.988-1.037) | 0.999(0.971-1.029) | 0.982(0.950-1.015) | 1.019(0.996-1.042) | 0.981(0.949-1.014) | 0.990(0.973-1.008) |
| 8 | 1.013(0.991-1.036) | 1.004(0.977-1.032) | 0.984(0.954-1.015) | 1.021(1.000-1.043) * | 0.971(0.941-1.001) | 0.996(0.980-1.013) |
| 9 | 1.015(0.994-1.037) | 1.011(0.985-1.038) | 0.990(0.961-1.019) | 1.024(1.004-1.045) * | 0.961(0.933-0.989) | 1.004(0.988-1.020) |
| 10 | 1.017(0.995-1.040) | 1.020(0.993-1.048) | 0.997(0.967-1.028) | 1.027(1.006-1.049) * | 0.950(0.922-0.979) | 1.014(0.997-1.031) |
| 11 | 1.019(0.993-1.046) | 1.030(0.997-1.063) | 1.007(0.971-1.044) | 1.031(1.003-1.057) * | 0.939(0.907-0.974) | 1.024(1.004-1.045) * |
| 12 | 1.022(0.988-1.056) | 1.041(1.000-1.084) | 1.017(0.972-1.065) | 1.035(1.003-1.068) * | 0.929(0.888-0.971) | 1.036(1.011-1.062) * |
| 13 | 1.024(0.982-1.068) | 1.052(1.000-1.107) | 1.029(0.972-1.089) | 1.038(0.998-1.080) | 0.918(0.868-0.971) | 1.048(1.016-1.080) * |
| 0-0 | 1.030(0.974-1.088) | 1.056(0.988-1.129) | 1.086(1.007-1.171) * | 1.020(0.968-1.075) | 1.034(0.965-1.109) | 1.028(0.986-1.072) |
| 0-1 | 1.055(0.960-1.159) | 1.096(0.978-1.229) | 1.147(1.009-1.303) * | 1.038(0.949-1.135) | 1.064(0.946-1.197) | 1.043(0.971-1.120) |
| 0-2 | 1.076(0.954-1.213) | 1.120(0.969-1.295) | 1.181(1.004-1.390) * | 1.054(0.941-1.181) | 1.088(0.937-1.263) | 1.044(0.953-1.144) |
| 0-3 | 1.093(0.954-1.253) | 1.130(0.958-1.333) | 1.193(0.991-1.436) | 1.070(0.941-1.217) | 1.105(0.933-1.309) | 1.036(0.934-1.150) |
| 0-4 | 1.109(0.956-1.285) | 1.132(0.947-1.354) | 1.188(0.971-1.452) | 1.086(0.944-1.249) | 1.114(0.926-1.340) | 1.023(0.913-1.146) |
| 0-5 | 1.123(0.959-1.314) | 1.130(0.933-1.368) | 1.172(0.946-1.453) | 1.103(0.950-1.280) | 1.113(0.912-1.358) | 1.008(0.893-1.139) |
| 0-6 | 1.136(0.961-1.343) | 1.127(0.919-1.380) | 1.151(0.916-1.446) | 1.121(0.957-1.314) | 1.102(0.891-1.364) | 0.995(0.874-1.132) |
| 0-7 | 1.150(0.963-1.374) | 1.126(0.907-1.397) | 1.130(0.887-1.440) | 1.143(0.966-1.351) | 1.081(0.860-1.358) | 0.985(0.859-1.131) |
| 0-8 | 1.166(0.966-1.406) | 1.131(0.900-1.421) | 1.112(0.860-1.438) | 1.167(0.977-1.394) | 1.049(0.823-1.338) | 0.982(0.848-1.136) |
| 0-9 | 1.183(0.971-1.442) | 1.143(0.898-1.455) | 1.101(0.839-1.444) | 1.195(0.991-1.441) | 1.008(0.779-1.304) | 0.986(0.845-1.150) |
| 0-10 | 1.203(0.977-1.481) | 1.166(0.905-1.501) | 1.098(0.825-1.459) | 1.228(1.009-1.494) * | 0.958(0.731-1.255) | 0.999(0.849-1.176) |
| 0-11 | 1.226(0.986-1.525) | 1.200(0.920-1.566) | 1.105(0.819-1.490) | 1.266(1.030-1.556) * | 0.900(0.678-1.194) | 1.023(0.862-1.215) |
| 0-12 | 1.253(0.994-1.578) | 1.249(0.943-1.655) | 1.124(0.819-1.543) | 1.310(1.053-1.630) * | 0.836(0.621-1.125) | 1.060(0.883-1.273) |
| 0-13 | 1.283(1.000-1.646) | 1.315(0.971-1.780) | 1.156(0.822-1.627) | 1.360(1.075-1.722) * | 0.767(0.558-1.056) | 1.111(0.911-1.353) |

*: *P < 0.05*

Abbreviations: CHD: coronary heart disease; CO: carbon monoxide.

Table S5 The association between an increase of 10ug/m^3^ of O_3_ and CHD admission in Hefei, 2014-2021

| Single-day | Relative risk (95%,confidence interval) | Multi-day | Relative risk (95%, confidence interval) |
| --- | --- | --- | --- |
| 0 | 0.993(0.988-0.999) * | 0-0 | 0.993(0.988-0.999) * |
| 1 | 0.996(0.993-0.999) * | 0-1 | 0.990(0.982-0.998) * |
| 2 | 0.998(0.996-1.001) | 0-2 | 0.988(0.979-0.997) * |
| 3 | 1.000(0.997-1.002) | 0-3 | 0.988(0.978-0.997) * |
| 4 | 1.000(0.998-1.002) | 0-4 | 0.988(0.977-0.997) * |
| 5 | 1.000(0.998-1.001) | 0-5 | 0.987(0.977-0.997) * |
| 6 | 0.999(0.996-1.001) | 0-6 | 0.986(0.976-0.996) * |
| 7 | 0.998(0.994-1.002) | 0-7 | 0.984(0.973-0.995) * |

*: *P < 0.05*

Abbreviations: CHD: coronary heart disease; O_3_: ozone

Table S6 The association between an increase of 10ug/m^3^ of O_3_ and CHD admission in different subgroups (relative risk and 95% confidence interval)

| lag | male | female | age<65yeras | age≥65years | hot season | cold season |
| --- | --- | --- | --- | --- | --- | --- |
| 0 | 0.994(0.987-1.001) | 0.992(0.984-1.001) | 0.997(0.988-1.006) | 0.992(0.985-0.998) | 0.997(0.992-1.003) | 0.991(0.983-0.999) * |
| 1 | 0.997(0.994-1.001) | 0.995(0.990-0.999) | 1.000(0.995-1.004) | 0.995(0.991-0.998) | 1.000(0.997-1.003) | 0.994(0.990-0.998) * |
| 2 | 1.000(0.997-1.002) | 0.997(0.993-1.000) | 1.001(0.998-1.005) | 0.997(0.994-1.000) | 1.002(1.000-1.004) | 0.996(0.993-0.999) * |
| 3 | 1.001(0.998-1.004) | 0.998(0.994-1.002) | 1.002(0.998-1.006) | 0.999(0.996-1.001) | 1.003(1.000-1.005) | 0.998(0.994-1.001) |
| 4 | 1.001(0.998-1.003) | 0.999(0.995-1.002) | 1.001(0.997-1.004) | 0.999(0.997-1.002) | 1.003(1.000-1.005) | 0.999(0.996-1.002) |
| 5 | 1.000(0.997-1.002) | 0.999(0.997-1.002) | 0.999(0.996-1.002) | 1.000(0.998-1.002) | 1.002(1.000-1.004) | 1.001(0.998-1.003) |
| 6 | 0.998(0.995-1.001) | 1.000(0.996-1.003) | 0.997(0.993-1.000) | 1.000(0.997-1.003) | 1.001(0.999-1.004) | 1.002(0.998-1.005) |
| 7 | 0.996(0.992-1.001) | 1.000(0.994-1.006) | 0.994(0.988-1.000) | 1.000(0.995-1.004) | 1.000(0.997-1.004) | 1.003(0.997-1.008) |
| 0-0 | 0.994(0.987-1.001) | 0.992(0.984-1.001) | 0.997(0.988-1.006) | 0.992(0.985-0.998) * | 0.997(0.992-1.003) | 0.991(0.983-0.999) * |
| 0-1 | 0.992(0.982-1.002) | 0.987(0.975-0.999) * | 0.996(0.983-1.010) | 0.987(0.977-0.996) * | 0.997(0.989-1.006) | 0.985(0.974-0.996) * |
| 0-2 | 0.991(0.980-1.002) | 0.984(0.971-0.997) * | 0.998(0.983-1.012) | 0.984(0.973-0.994) * | 0.999(0.990-1.008) | 0.981(0.968-0.993) * |
| 0-3 | 0.992(0.980-1.004) | 0.982(0.968-0.995) * | 0.999(0.984-1.015) | 0.982(0.971-0.993) * | 1.002(0.992-1.011) | 0.978(0.965-0.992) * |
| 0-4 | 0.992(0.981-1.005) | 0.980(0.966-0.995) * | 1.000(0.984-1.016) | 0.982(0.970-0.993) * | 1.004(0.994-1.014) | 0.978(0.964-0.992) * |
| 0-5 | 0.992(0.980-1.005) | 0.980(0.965-0.995) * | 0.999(0.983-1.016) | 0.981(0.970-0.993) * | 1.007(0.996-1.017) | 0.978(0.964-0.993) * |
| 0-6 | 0.990(0.978-1.003) | 0.980(0.965-0.995) * | 0.996(0.979-1.013) | 0.981(0.969-0.993) * | 1.008(0.997-1.019) | 0.980(0.965-0.996) * |
| 0-7 | 0.987(0.973-1.001) | 0.979(0.963-0.996) * | 0.990(0.972-1.008) | 0.981(0.968-0.994) * | 1.008(0.996-1.020) | 0.983(0.965-1.000) |

*: *P < 0.05*

Abbreviations: CHD: coronary heart disease; O_3_: ozone

Figure S1 Spearman's correlation coefficients between the different meteorological factors and air pollutants.


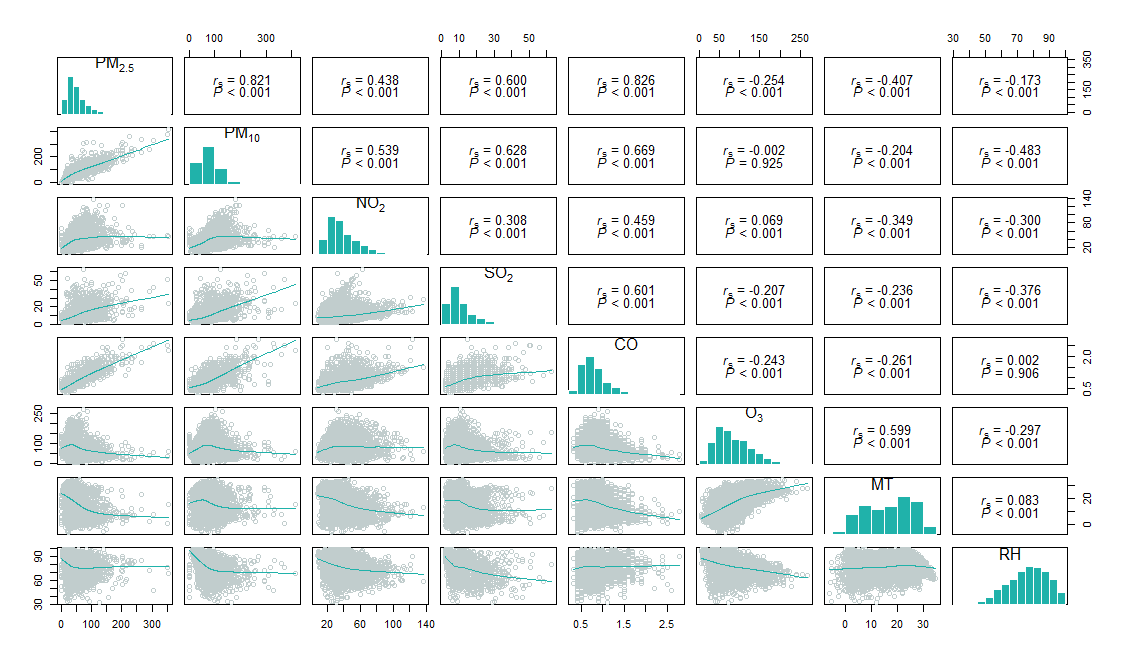


Figure S2 Concentration-response relationship curves between NO_2_, CO and O_3_ exposure levels and daily hospitalization in patients admitted with CHD.


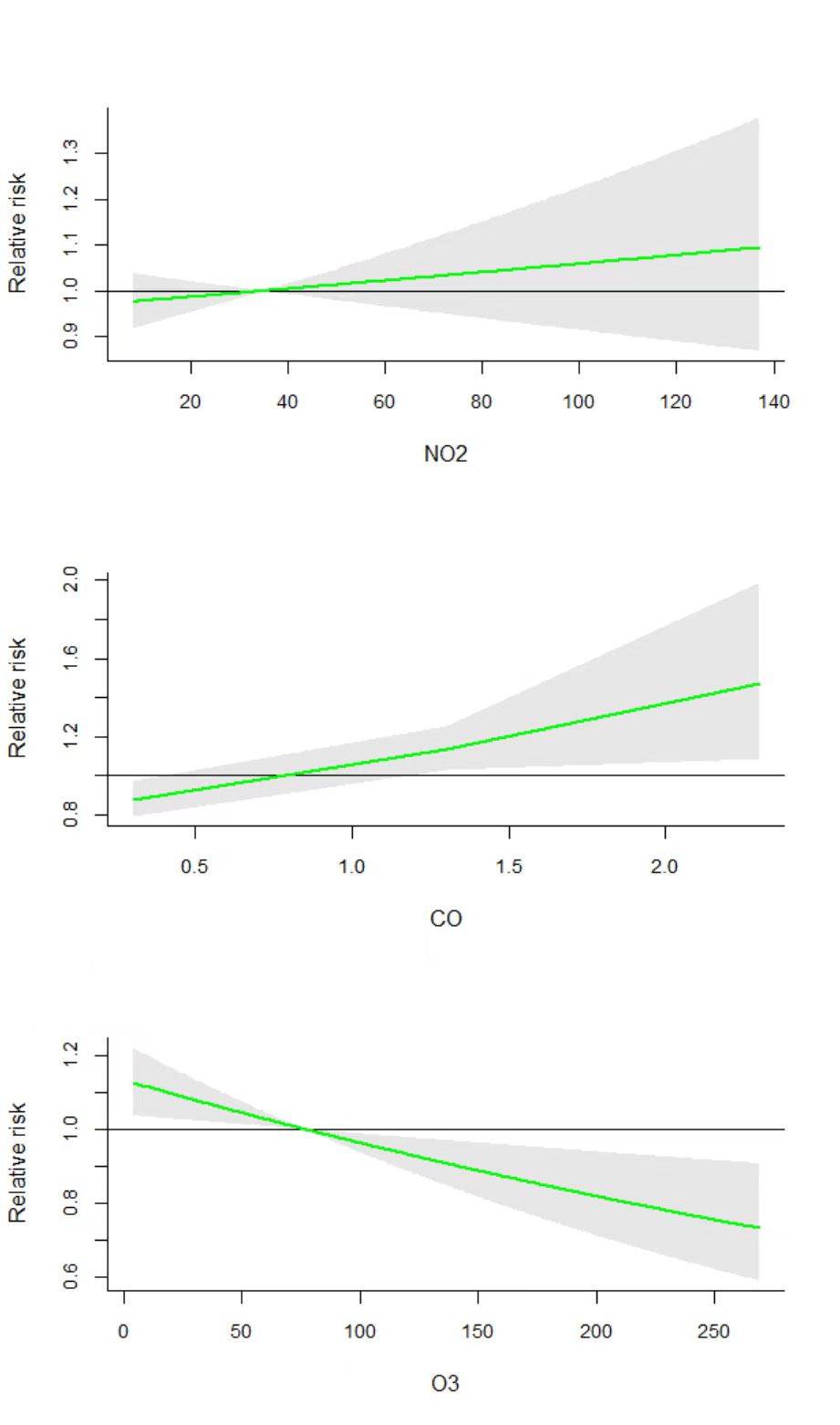


Figure S3 The single-day association between an increase of 10ug/m^3^ of NO_2_ and CHD when varying the degree of freedom (3-5dfs) for MT, RH and the df (6-8dfs/year) for time.


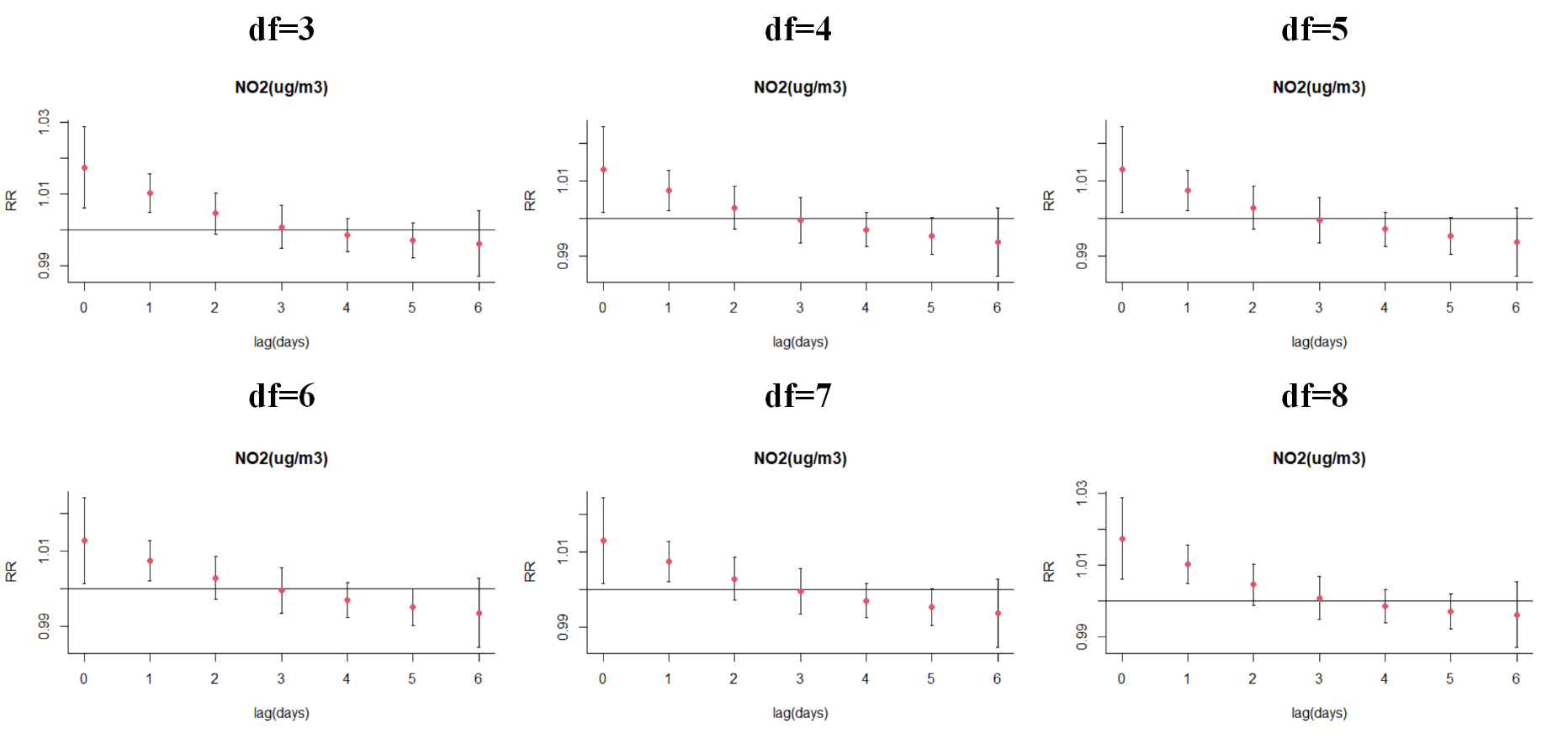


Figure S4 The single-day association between an increase of 1ug/m^3^ of CO and CHD when varying the degree of freedom (3-5dfs) for MT, RH and the df (6-8dfs/year) for time.


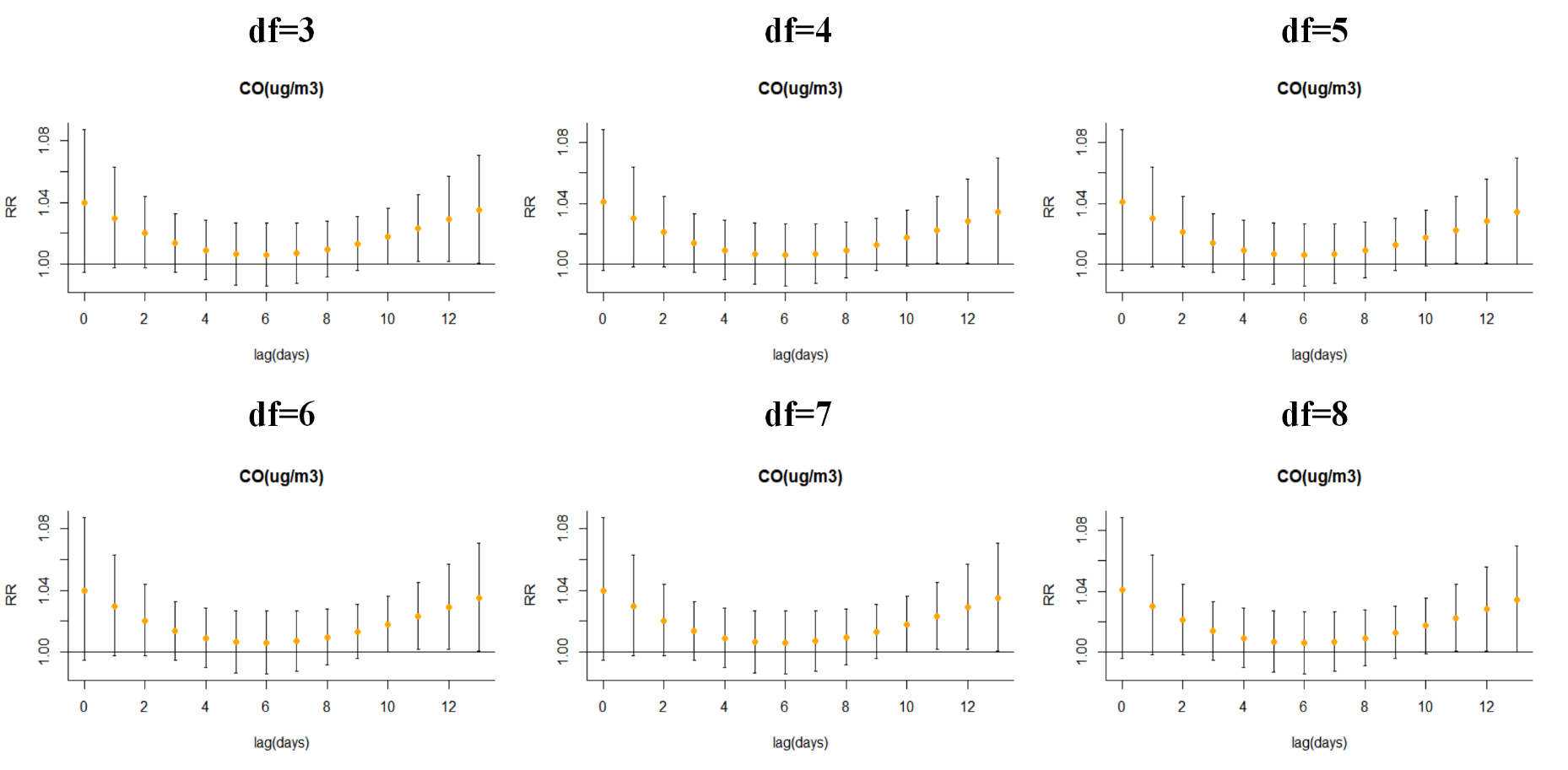


Figure S5 The single-day association between an increase of 10ug/m^3^ of O_3_ and CHD when varying the degree of freedom (3-5dfs) for MT, RH and the df (6-8dfs/year) for time.


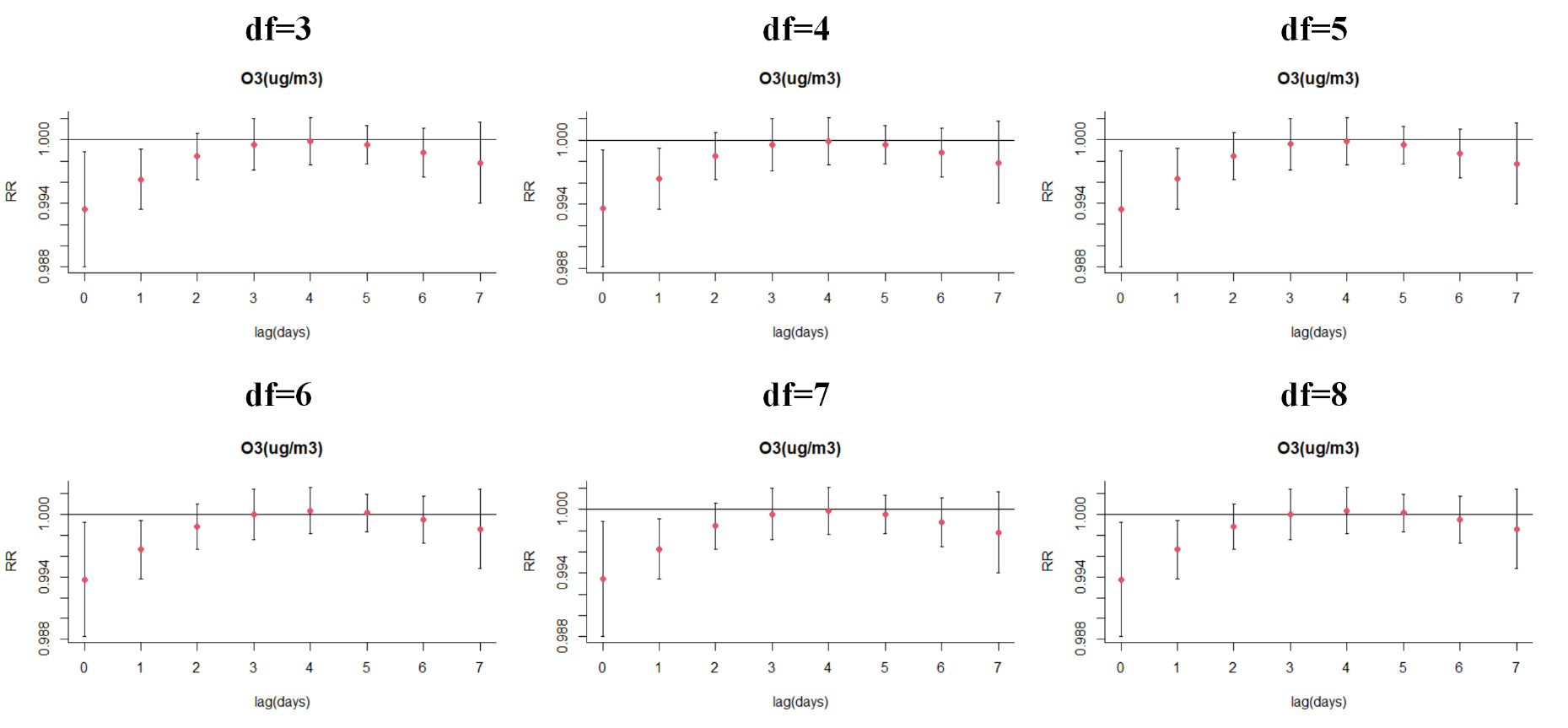

Supplement: Supplementary file 1 [file Data_Sheet_1.docx]
